# “The Octet”: Eight Protein Kinases that Control Mammalian DNA Replication

**DOI:** 10.3389/fphys.2012.00368

**Published:** 2012-09-26

**Authors:** Melvin L. DePamphilis, Christelle M. de Renty, Zakir Ullah, Chrissie Y. Lee

**Affiliations:** ^1^Program in Genomics of Differentiation, National Institute of Child Health and Human Development, National Institutes of HealthBethesda, MD, USA; ^2^Department of Biology, Syed Babar Ali School of Science and Engineering, Lahore University of Management SciencesLahore, Pakistan

**Keywords:** cyclin-dependent kinases, cyclins, checkpoint kinases, mitotic cell cycle, endoreplication, DNA re-replication, Cdc7, Dbf4

## Abstract

Development of a fertilized human egg into an average sized adult requires about 29 trillion cell divisions, thereby producing enough DNA to stretch to the Sun and back 200 times (DePamphilis and Bell, 2011)! Even more amazing is the fact that throughout these mitotic cell cycles, the human genome is duplicated once and only once each time a cell divides. If a cell accidentally begins to re-replicate its nuclear DNA prior to cell division, checkpoint pathways trigger apoptosis. And yet, some cells are developmentally programmed to respond to environmental cues by switching from mitotic cell cycles to endocycles, a process in which multiple S phases occur in the absence of either mitosis or cytokinesis. Endocycles allow production of viable, differentiated, polyploid cells that no longer proliferate. What is surprising is that among the 516 (Manning et al., 2002) to 557 (BioMart web site) protein kinases encoded by the human genome, only eight regulate nuclear DNA replication directly. These are Cdk1, Cdk2, Cdk4, Cdk6, Cdk7, Cdc7, Checkpoint kinase-1 (Chk1), and Checkpoint kinase-2. Even more remarkable is the fact that only four of these enzymes (Cdk1, Cdk7, Cdc7, and Chk1) are essential for mammalian development. Here we describe how these protein kinases determine when DNA replication occurs during mitotic cell cycles, how mammalian cells switch from mitotic cell cycles to endocycles, and how cancer cells can be selectively targeted for destruction by inducing them to begin a second S phase before mitosis is complete.

## Introduction

The mitotic cell cycle restricts duplication of mammalian nuclear DNA to once and only once each time the cell divides. Re-replication of regions of nuclear DNA that have already been replicated prior to completion of S phase (termed “DNA re-replication”) is an aberrant form of the mitotic cell cycle that ends in cell death. However, some cells are developmentally programmed to exit their mitotic cell cycle in order to undergo multiple rounds of nuclear genome duplication without an intervening mitosis (termed “endoreplication”). Here we describe the characteristics of each of these replication patterns and the roles played by the eight protein kinases that act directly to link DNA replication to cell division and to restrict duplication of the nuclear genome to once and only once per cell division.

### The mitotic cell cycle

**Figure 1:**
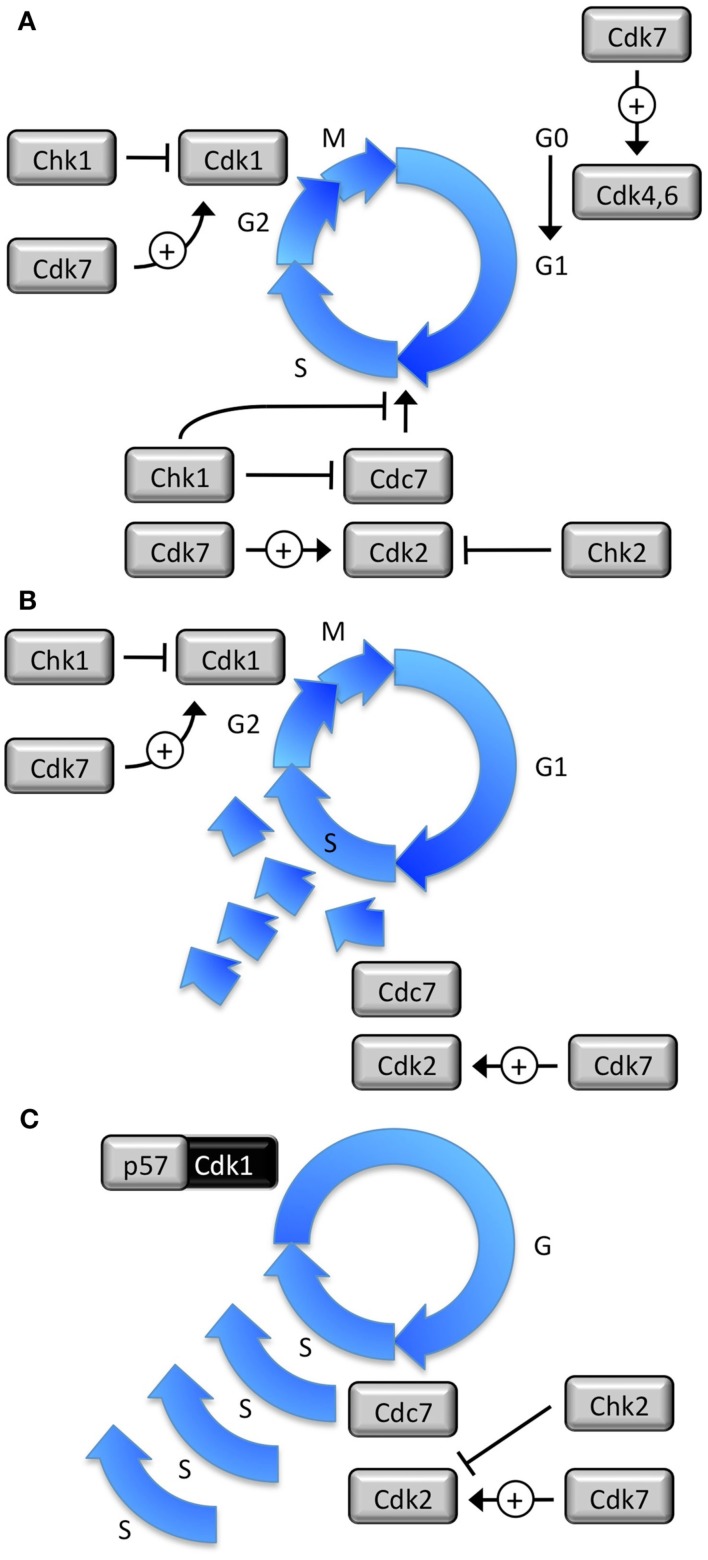
**Eight protein kinases regulate DNA replication in mammalian cells**. **(A)** Mitotic cell cycles produce one and only one copy of the nuclear genome per cell division. Cdk7•CcnH activates Cdk1, Cdk2, Cdk4, and Cdk6. Cdk4•CcnD and Cdk6•CcnD drives cells from a quiescent state (G0) into a proliferative state. Cdk1•CcnB1 triggers mitosis. Cdc7•Dbf4 and Cdk2•CcnE initiate DNA replication. Cdk2•CcnA2 prevents DNA re-replication. Cdk1•CcnA2 prevents premature initiation during G2 phase. Chk1 inhibits Cdk1 and Cdk2 in response to either stalled replication forks or DNA damage. Chk2 inhibits Cdc7 and Cdk2 in response to double strand DNA breaks. **(B)** If proliferating cells attempt to re-replicate their nuclear DNA before exiting the mitotic cell cycle, the result is a random accumulation of partially re-replicated chromosomes that generates cells with >4C but <8C DNA content that undergo apoptosis. **(C)** Endocycles produce multiple copies of the nuclear genome in the absence of cell division. In trophoblast giant cells, Cdk2 is essential for DNA replication in the absence of Cdk1 activity, which is inhibited by p57, and Chk1 is selectively suppressed. Cdc7 and Cdk7 are essential for initiation of preIC assembly. Cdk4 and Cdk6 are assumed to be irrelevant.

The mitotic cell cycle is a sequence of events in which the nuclear DNA of eukaryotic cells is duplicated once and only once each time a cell divides in order to produce two diploid cells, both of which contain two complete copies of each chromosome (2C DNA content). In mitotic cell cycles, nuclear DNA replication is linked to cell division, and its copy number is strictly regulated, whereas mitochondrial DNA replication is not linked to cell division and its copy number varies widely among cell types (DePamphilis and Bell, 2011). Nuclear DNA replication is controlled directly by only eight protein kinases. Most cells in adult mammals exist in a quiescent state termed G0, from which they can reenter their mitotic cell cycle through the action of two cyclin-dependent protein kinases (CDK) termed Cdk4 and Cdk6 (Figure 1A; DePamphilis and Bell, 2011). Two other protein kinases, Cdc7 and Cdk2, then drive mammalian cells from a period of cell growth (G1 phase) into a period of nuclear DNA replication (S phase). Upon completion of DNA replication (G2 phase), a fifth protein kinase (Cdk1) drives the cell into mitosis while simultaneously inhibiting initiation of the next S phase by phosphorylating at least one of the proteins required to initiate DNA replication. However, neither Cdk1 nor Cdk2 are active unless phosphorylated by Cdk7 at one site and dephosphorylated by a Cdc25 protein phosphatase at a different site. Cdc25 activity is suppressed when it is phosphorylated by Checkpoint kinase-1 (Chk1), a protein kinase that is activated by either stalled replication forks or damage in one of the DNA strands. Thus, Chk1 prevents cells from entering mitosis until nuclear DNA replication is complete. Chk1 also blocks initiation of DNA replication in response to replication stress. Another closely related protein kinase, Checkpoint kinase-2 (Chk2), prevents cells with double strand breaks in their nuclear DNA from entering S phase, a condition that would result in cell death.

### The four protein kinases that drive the mitotic cell cycle

**Figure 2:**
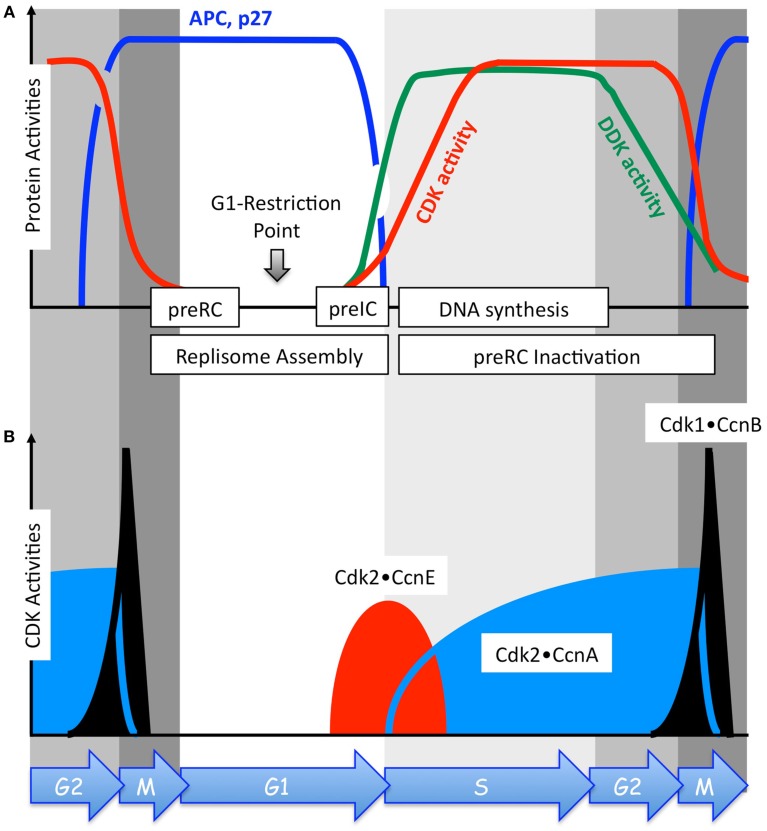
**The activities of DDK, CDKs, and APC, and the level of the CDK-specific inhibitor p27 oscillate during continuous proliferation of human cells in culture**. **(A)** Top panel relates changes in the activities of the anaphase-promoting complex (APC, blue line), DDK (Dbf4-dependent Cdc7 kinase, green line), and total CDKs (red line) as a function of the phase of the mitotic cell cycle. The cellular level of the CDK-specific inhibitor p27 (blue line) parallels changes in APC activity. **(B)** Bottom panel relates the relative changes that occur in the activities of Cdk1•CcnB (black), Cdk2•CcnE (red), and Cdk2•CcnA (blue) as a function of the phase of the mitotic cell cycle. These changes result from changes in the levels of the indicated cyclin protein, not the CDK catalytic subunit. Cyclin expression is regulated through cyclin stability rather than through transcription or translation (Morgan, 2007). Cyclins are targeted for ubiquitin-dependent degradation by a series of feedback loops involving CDKs and ubiquitin ligases whose activities are themselves cell cycle dependent (Figure 3). As cells exit metaphase, the APC targets CcnA and CcnB for ubiquitin-dependent degradation. Therefore, the combined effects of the APC, p27, and the protein kinase Wee1 (not shown) inactivate Cdk1, Cdk2, Cdk4, and Cdk6. PreRC assembly (Figure 5, top panel) occurs only in the absence of CDK activity, the period encompassed by anaphase, telophase, and G1 phase (Figure 2). PreIC assembly (Figure 5) begins with the appearance of DDK and CDK activities in late G1 phase **(A)**. PreRC components are inactivated during S and G2 phases in order to prevent DNA re-replication (Figure 6). The G1-Restriction Point occurs after preRC assembly and before preIC assembly (Wu and Gilbert, 1997).

**Figure 3:**
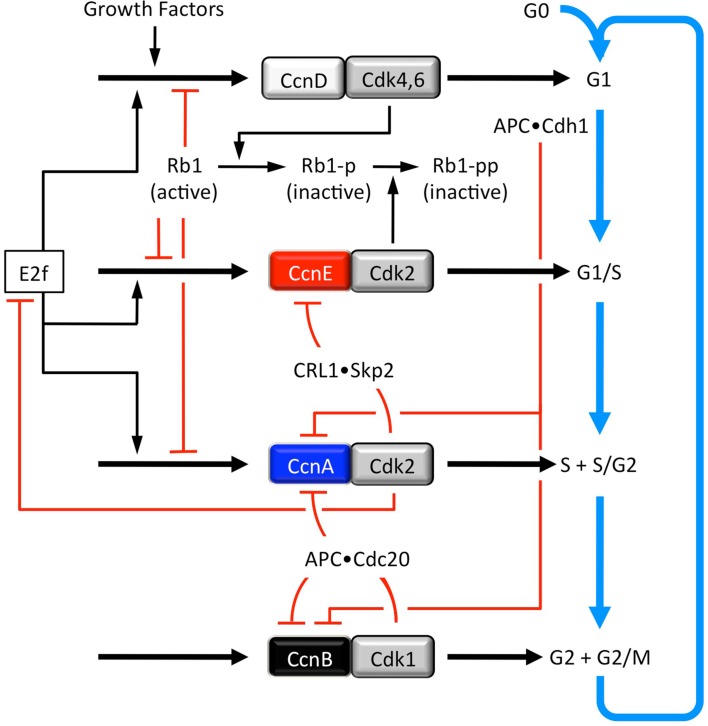
**Four CDK•cyclin activities regulate progress through successive phases of the mitotic cell cycle (adapted from Gerard and Goldbeter, 2009)**. Growth factors activate the synthesis of the transcription factor Ap1, which promotes synthesis of CcnD, which forms a reversible complex with Cdk4 and Cdk6 (Cdk4,6). The activity of Cdk4,6•CcnD, like that of Cdk1 and Cdk2, is regulated by phosphorylation. Cdk4,6•CcnD phosphorylates and thereby inactivates the retinoblastoma tumor suppressor (Rb1). This phosphorylation allows activation of the transcription factor E2f. The non-phosphorylated and monophosphorylated forms of Rb1 can form a complex with E2f, thereby inhibiting its transcriptional activity. A second phosphorylation of Rb1 by Cdk2•CcnE completely inhibits Rb1, thereby fully activating E2f. E2f up-regulates the synthesis of CcnD, CcnE, and CcnA. CcnE synthesis is induced by E2f and inhibited by Rb1-p and Rb1-pp. CcnE reversibly forms a complex with Cdk2, the activity of which is regulated by phosphorylation and dephosphorylation (Figure 3) and through association with the CDK-specific inhibitors p21 and p27 (Figure 2A). As Cdk2•CcnE accumulates, it phosphorylates p21 and p27, making them targets for ubiquitination by CRL1•Skp2. Therefore, this phosphorylation event results in ubiquitin-dependent degradation of p21 and p27 by the 26S proteasome. Similarly, phosphorylation of CcnE by Cdk2•CcnA results in ubiquitin-dependent degradation of CcnE by the 26S proteasome, thereby ending the activation of preinitiation complexes at replication origins. As with Cdk2•CcnE, Cdk2•CcnA is regulated by phosphorylation and dephosphorylation (Figure 3) as well as by p21 and p27. Cdk2•CcnA controls the S to G2 phase transition by eliciting the phosphorylation and subsequent degradation of E2f. Both Cdk2•CcnA and Cdk1•CcnB phosphorylate and thereby inhibit Cdh1, a protein that allows the APC ubiquitin ligase to target cyclins A and B during the anaphase through G1 phase period when CDK activity is low. This results in the accumulation of CcnA and CcnB. As Cdk2•CcnA activity accumulates, it phosphorylates and thereby inhibits Cdh1, thus leading to the eventual transition from G2 to M phase. CcnB reversibly forms a complex with Cdk1, and Cdk1•CcnB also phosphorylates and inhibits Cdh1, which amounts to a positive feedback loop for production of Cdk1•CcnB. As with Cdk2•CcnE and Cdk2•CcnA, Cdk1•CcnB activity is regulated by phosphorylation and dephosphorylation (Figure 3) as well as p21. At its peak, Cdk1•CcnB activity initiates entry into mitosis. It also activates, by phosphorylation, Cdc20, which creates a negative feedback loop wherein the APC•Cdc20 targets CcnA and CcnB for ubiquitin-dependent degradation. As a result, the levels of Cdk2•CcnA and Cdk1•CcnB activity decrease, thereby allowing cells to complete mitosis and undergo cytokinesis. In the presence of sufficient amounts of GF, cells spontaneously begin a new mitotic cell cycle, corresponding to a new round of oscillations.

Of the eight protein kinases that directly link nuclear DNA replication to cell division (Figure 1A), only Cdk1 and Cdk2 exhibit dramatic oscillations in their activity during continuous mitotic cell cycles (Figure 2B). Changes in Cdk4 and Cdk6 activity are dramatic only when cells transition from G0 to S phase and back again. Nevertheless, the oscillations observed in the activities of four CDK•cyclin protein complexes are sufficient to create a computer model for the mitotic cell cycle in mammals (Gerard and Goldbeter, 2009). These activities are linked together through a succession of both positive and negative feedback loops that produce a self-sustained biochemical oscillator that drives the cell cycle in one direction (Figure 3).

In response to a growth factor stimulus, cyclin D (CcnD) levels increase and Cdk4•CcnD and Cdk6•CcnD drive the cell from G0 to G1 phase by phosphorylating the retinoblastoma tumor suppressor protein (Rb1). In this capacity, their activities are redundant and therefore considered as a single CDK•cyclin activity. Since phosphorylated forms of Rb1 inhibit transcription factor E2f, inactivation of Rb1 allows E2f to up-regulate expression of a large number of gene activities that are required for DNA replication. Among these are cyclins E (CcnE), A (CcnA), and B (CcnB). However, only CcnE accumulates during G1 phase, because the anaphase-promoting complex (APC) associated with Cdh1, one of the two APC regulatory subunits, ubiquitinates CcnA and CcnB, thereby targeting them for degradation by the 26S proteasome (reviewed in Pesin and Orr-Weaver, 2008). The APC•Cdh1 is active only when Cdk1 and Cdk2 activities are suppressed (i.e., from anaphase in mitosis through G1 phase; Figure 2A).

Cell cycle dependent changes in the levels of cyclin proteins, not their catalytic subunits, create corresponding changes in CDK activities (Figure 2B) that drive the mitotic cell cycle. In response to increasing levels of CcnE, Cdk2•CcnE activity drives the cell from G1 into S phase (discussed below). In response to increasing levels of cyclin A, Cdk2•CcnA phosphorylates and inactivates CcnE by making it a target for the ubiquitin ligase CRL1•Skp2, thereby inhibiting further activation of DNA replication complexes at replication origins. Cdk2•CcnA also phosphorylates and inactivates E2f, thereby down-regulating expression of genes required for DNA replication, as well as several proteins that are essential for assembly of prereplication complexes (preRCs), thereby preventing re-licensing of replication origins during S phase (discussed below). In response to increasing levels of CcnB, a Cdk1•CcnB complex forms in the cytoplasm where it initiates conformational changes that allow Cdk1 to alter its phosphorylation status and become an active kinase (reviewed in Pines, 2006). The active Cdk1•CcnB complex then translocates to the nucleus where it begins phosphorylating nuclear substrates that are necessary to initiate mitosis.

**Figure 4:**
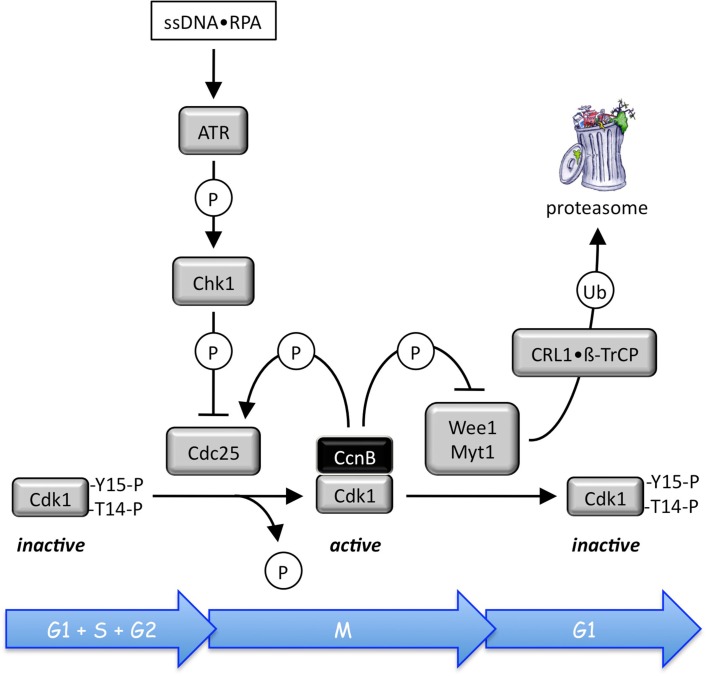
**Site specific phosphorylation and dephosphorylation events regulate CDK•cyclin activity (reviewed in Domingo-Sananes et al., 2011)**. Human CDK•cyclin complexes are maintained in an inactive state through Wee1 phosphorylation of Tyr-15, and Myt1 phosphorylation of both Tyr-15 and Thr-14 adjacent to the CDK catalytic site. For example, prophase begins when the stockpile of Thr-14 and Tyr-15 phosphorylated Cdk1•CcnB is abruptly activated by Cdc25-mediated dephosphorylation of Cdk1. As Cdk1•CcnB activity appears, it catalyzes its own activation by two feedback loops. Wee1 is phosphorylated by Cdk1•CcnB, facilitating its degradation by the ubiquitin ligases CRL1•βTrCP and CRL1•Tome1. Conversely, Cdc25 is activated by multiple CDK phosphorylations. This allows Cdk1•CcnB to convert graded inputs into switch-like, irreversible responses once a critical portion of the enzyme becomes active. In mammalian cells, all three Cdc25 isoforms have been implicated in cell cycle regulation. Cdc25A regulates both G_1_/S and S/G_2_ transitions, while Cdc25B and Cdc25C regulate late G_2_ and mitosis, respectively (Lavecchia et al., 2010).

The activities of each of these CDK•cyclin complexes are regulated through positive feedback loops involving phosphorylation and dephosphorylation by Cdc25, Wee1, and Myt1 (Figure 4). In addition, negative feedback loops regulate Cdk1 and Cdk2 activities through APC ubiquitination of CcnA and CcnB (Figure 3). However, during prometaphase, Cdc20 activates the APC, because Cdc20 is active only when phosphorylated by either Cdk1 or Cdk2, whereas Cdh1 is inactive when phosphorylated. During S and G2 phases, the APC-specific inhibitor Emi1 prevents phosphorylation of Cdc20 by Cdk2, and Cdk2•CcnA phosphorylation of the APC inhibits binding of Cdh1 to the APC and targets Cdh1 for ubiquitination by CRL1/SCF•Skp2 and subsequent degradation by the 26S proteasome. Emi1 is selectively degraded during prometaphase. In addition, both Cdk1 and Cdk2 (but not Cdk4 and Cdk6) are regulated through their reversible association with the CDK-specific inhibitors Cdkn1a/p21/Cip1/and Cdkn1b/p27/Kip1, two proteins whose activities also are regulated by feedback loops involving CDK-dependent phosphorylation and ubiquitin-dependent degradation (reviewed in Guardavaccaro and Pagano, 2006). Cell cycle dependent expression of both p21 and p27 follows that of APC activity (Figure 2A). However, it is the CDK•cyclin network itself that controls the balance between Rb1 and E2f through CDK-dependent phosphorylation events, and it is this network alone that can account for the periodic oscillations that drive the mitotic cell cycle (Gerard and Goldbeter, 2011).

### DNA re-replication

DNA re-replication (Figure 1B) is an aberrant event that occurs during mitotic cell cycles either when drugs are used to interfere with DNA replication or mitosis, or when genes that regulate initiation of DNA replication are suppressed or over-expressed. The result is a population of cells whose DNA content is >4C but generally less than 8C. This excess DNA replication is a consequence of incomplete chromosome reduplication that results in stalled replication forks and DNA damage. Thus, instead of a viable aneuploid cell, DNA re-replication results in DNA damage and stalled replication forks that induce the cell’s DNA damage response (activation of the ATR → Chk1 → Cdc25 pathway; Figure 4) and eventually apoptosis (Niida et al., 2005; Lin and Dutta, 2007; Liu et al., 2007). DNA re-replication is prevented during mitotic cell cycles by the combined effects of Cdk2•CcnA, CRL4•Cdt2, Geminin, and Chk1 (discussed below).

### Developmentally programmed polyploidy

Remarkably, some cells are developmentally programmed to exit their mitotic cell cycle in response to environmental signals and then differentiate into non-proliferating, viable, polyploid cells. This developmentally programmed polyploidy is a normal part of animal and plant development that occurs frequently in ferns, flowering plants, arthropods, fish, and salamanders, but rarely in mammals. Developmentally programmed polyploidy occurs by four different mechanisms (Ullah et al., 2009a; Lacroix and Maddox, 2012). (1) “Acytokinetic mitosis” refers to cells that become multinucleated by failing to undergo cytokinesis after mitosis. Examples are proliferating cells in the syncytial blastoderm of *Drosophila* embryos, in hepatocytes in the postnatal liver of mammals, and in cardiomyocytes proliferating during prenatal development. (2) “Cell fusion” refers to G1 phase cells that fuse together to form multinucleated, terminally differentiated, cells. Examples are differentiation of skeletal muscle myoblasts into myotubes, monocytes into osteoclasts, and formation of placental syncytiotrophoblasts. (3) “Endomitosis” refers to cells that exit their mitotic cell cycle during anaphase and then undergo multiple S phases, each one terminated in anaphase. This results in a cell with a single giant nucleus that may subsequently fragment into a multinuclear appearance. Endomitosis occurs when megakaryoblasts differentiate into megakaryocytes (Bluteau et al., 2009), and in some plant cells (Weingartner et al., 2004). (4) “Endoreplication” (Figure 1C) refers to cells that exit the mitotic cell cycle during the G2 to M transition, conditions that allow multiple rounds of nuclear genome duplication in the absence of an intervening mitosis and cytokinesis (Edgar and Orr-Weaver, 2001; Lilly and Duronio, 2005; Lee et al., 2009). The result is a non-proliferating cell undergoing an alternating sequence of S and G phases, as outlined in Figure 8. Endoreplication is the primary mechanism for developmentally programmed polyploidy in arthropods, plants, differentiation of mammalian trophoblasts into giant cells, and possibly stress induced polyploidy in cardiomyoblasts, basal epithelial cells, and primitive podocytes. The term endocycles refers to any cell undergoing multiple S phases without completing mitosis.

### Endoreplication

Endoreplication occurs in eukaryotes when cells exit their mitotic cell cycle during the G2 to M phase transition, an event that can be mimicked in yeast, flies, and mammals by inhibition of Cdk1 activity (Ullah et al., 2009b). Endoreplication is distinguished from DNA re-replication in three ways. Developmentally programmed polyploidy produces cells with >4C DNA content, but whose DNA content is an integral multiple of 2C (e.g., 4C, 8C, 16C, etc.), whereas DNA re-replication does not. Second, developmentally programmed polyploidy produces terminally differentiated cells that no longer proliferate, but remain viable. In contrast, DNA re-replication results in cell death. Finally, developmentally programmed polyploidy results in cells with either a single giant nucleus or multiple nuclei of normal size, whereas DNA re-replication produces cells with either an aneuploid nucleus or micronuclei in a process termed “mitotic slippage” (Riffell et al., 2009; Lee et al., 2010). Terminally differentiated polyploid cells may serve to regulate tissue size or organization, to trigger cell differentiation or morphogenesis, to increase the number of genes dedicated to tissue specific functions without increasing the number of cells, or to adapt to environmental conditions. Nevertheless, polyploidy is not always irreversible. Some polyploid cells in plants and insects have been shown to reenter a mitotic cell cycle (Fox et al., 2010), and DNA damaged polyploid cancer cells can reverse to diploidy at a low frequency (Martin et al., 2009).

## Genome Duplication

Although the mechanistic details of DNA replication are most well documented in yeast and frog egg extracts, the fact that mammals encode homologs or orthologs of each of the proteins required to initiate nuclear DNA replication clearly supports an evolutionarily conserved process that is essentially the same throughout the eukarya (Table 1).

**Table 1:** **Proteins required for replisome assembly in eukaryotes**.

*S. cerevisiae*	*Homo sapiens*
**PREREPLICATION COMPLEX**	
ORC(1–6)	ORC(1–6)
Cdc6	Cdc6
Cdt1	Cdt1
Mcm(2–7)	Mcm(2–7)
	HBO1
**PREINITIATION COMPLEX**	
Mcm(2–7)	Mcm(2–7)
sCdc45	Cdc45
Sld3	Treslin
Sld2	RecQ4
Dpb11	TopBP1
GINS	GINS
Pol-ε	Pol-ε
Mcm10	Mcm10
RPA	RPA
**REPLISOME**	
Mcm(2–7)	Mcm(2–7)
Cdc45	Cdc45
GINS	GINS
Pol-ε	Pol-ε
Mcm10	Mcm10
RPA	RPA
Pol-α:primase	Pol-α:primase
Pol-δ	Pol-δ

### Initiating nuclear DNA replication

Initiation of DNA replication in eukaryotic cells involves a DNA locus (the “replication origin”) at which a protein complex termed the “preRC” is assembled and then subsequently converted into a “preinitiation complex” (preIC). This last step permits DNA unwinding which is linked to DNA synthesis of the new complementary strands in both directions from the replication origin. Assembly of preRCs on chromatin is referred to as “replication licensing,” and the collection of proteins that carries out DNA replication at a replication fork is termed the “replisome” (detailed in DePamphilis and Bell, 2011).

Licensing of replication origins does not require a protein kinase activity; on the contrary, preRC assembly occurs only when CDK activity is suppressed (Schwob and Labib, 2006; Diffley, 2011), an event that is restricted to the period from mitotic anaphase until the G1 to S phase transition (Noguchi et al., 2006; Baldinger and Gossen, 2009; Figure 2). Origin licensing begins with assembly of a DNA helicase loader onto the replication origin. The DNA helicase loader consists of the six heterotypic subunits of ORC(1–6), Cdc6, and Cdt1 (Sivaprasad et al., 2006; Diffley, 2011). Orc1, ORC(2–5), and Orc6 are transported independently to the nucleus where they assemble into a stable ORC(1–6) complex that recruits Cdc6 and Cdt1 (Ghosh et al., 2011). This event is followed by assembly of the six heterotypic subunits of the MCM helicase [Mcm(2–7)] to form a “double hexamer” in which two MCM helicases are loaded onto opposite strands of the DNA duplex. One MCM helicase encompasses the “Watson” strand, and one encompasses the “Crick” strand. However, the MCM helicase remains inactive at this step. Since inefficient origin licensing increases the probability of stalled replication forks triggering a DNA damage response and apoptosis, the mutations in Orc1, Orc4, Orc6, Cdt1, and Cdc6 associated with individuals exhibiting microtia, patellar aplasia/hypoplasia, and short stature (the Meier-Gorlin syndrome; de Munnik et al., 2012) are the likely cause of this genetic defect.

**Figure 5:**
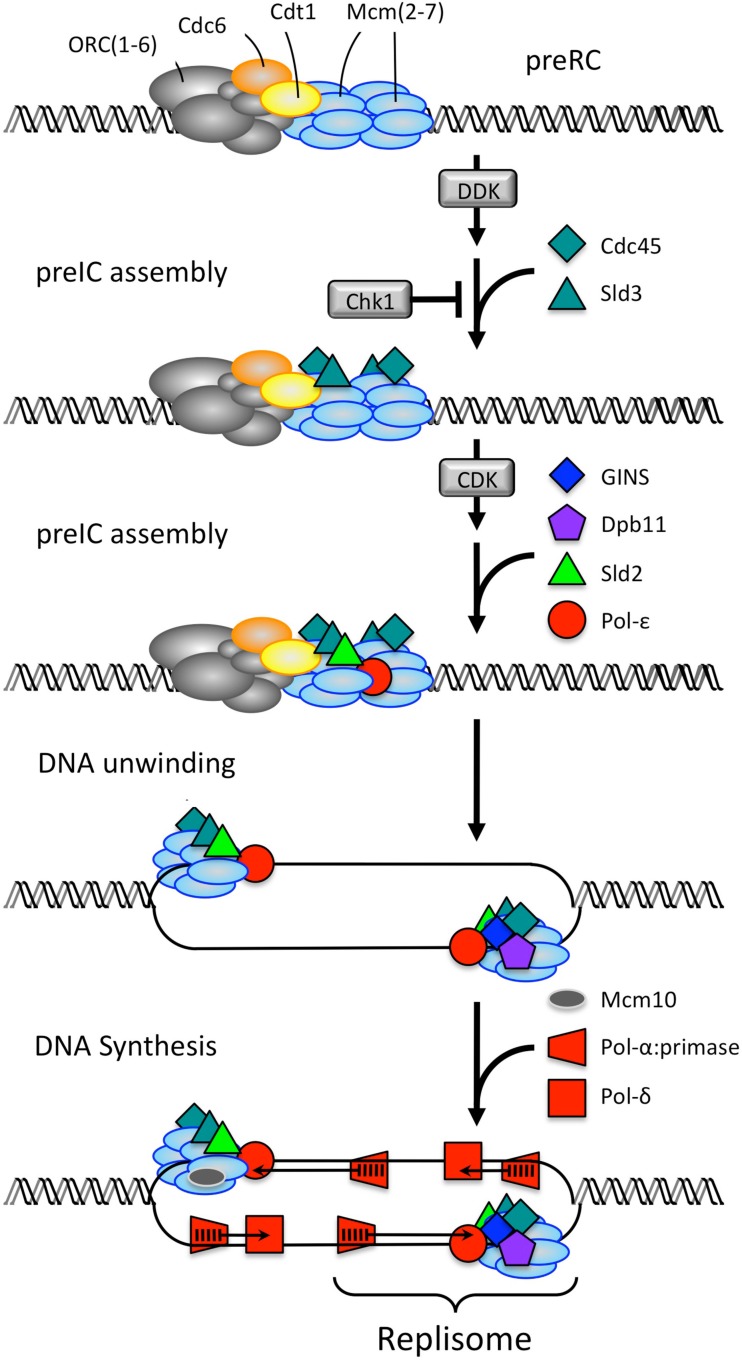
**Converting a preRC into two replisomes**. In *S. cerevisiae* (Heller et al., 2011), MCM/Mcm2-7 helicase is loaded onto origin DNA in an inactive form during the anaphase to G1 phase transition of the mitotic cell cycle to produce a prereplication complex. DDK (Cdc7•Dbf4 kinase) phosphorylates the MCM helicase during the G1 to S phase transition, thereby allowing recruitment of Sld3 and Cdc45 proteins. The S phase CDK (Cdk1•Clb5 or Cdk1•Clb6) phosphorylates Sld2 and Sld3, thereby triggering recruitment of Sld2, Dpb11, GINS, and Pol-ε. This event activates the MCM helicase, which in the presence of the single-strand DNA binding protein RPA, can unwind origin DNA. This event is followed by recruitment of Mcm10 (not shown). The formation of a Cdc45-MCM-GINS complex activates the helicase, which unwinds the DNA at the replication origin. Pol-α:primase and Pol-δ are loaded onto the unwound DNA to complete replisome assembly. Three genes have different names for the human analog of the indicate yeast protein: Treslin/Sld3, RecQ4/Sld2, and TopBP1/Dpb11.

Initiation of DNA replication is triggered by the Dbf4-dependent Cdc7 kinase (DDK) followed by a CDK activity (Heller et al., 2011) that converts the inactive MCM helicase in the preRC into an active helicase that can unwind origin DNA (Figure 5; Table 1). DNA unwinding is a prerequisite for DNA synthesis. The critical step is formation of a Cdc45•MCM•GINS complex that confers on the MCM helicase the ability to unwind the origin DNA (Ilves et al., 2010; Labib, 2010). The transition from preRC to replisome begins when DDK phosphorylates the Mcm4 and Mcm6 subunits of the MCM helicase (Randell et al., 2010; Sheu and Stillman, 2010), two events that are essential for assembly of Sld2 and Cdc45 proteins onto the preRC. The next step requires the phosphorylation of Sld2 and Sld3 by a CDK activity (Tanaka et al., 2007; Zegerman and Diffley, 2007). Single cell eukarya such as yeast use their only CDK (Cdk1) together with an S phase cyclin to carry out this step, whereas multicellular eukarya use Cdk2•CcnE. The phosphorylated forms of Sld2 and Sld3 bind Dpb11 protein, and the Sld2•Sld3•Dpb11 complex allows recruitment of the heterotypic four-subunit GINS complex. This event is essential for subsequent recruitment of Pol-ε, the DNA polymerase that extends the RNA-primed nascent DNA strand that initiates DNA synthesis on the leading strand template (Muramatsu et al., 2010). This protein complex constitutes a preIC with an active MCM helicase. The single-strand DNA binding protein RPA also is required to facilitate DNA unwinding. Finally, two active replisomes form upon addition of Pol-α:primase (the DNA polymerase that initiates RNA-primed DNA synthesis) and Pol-δ (the DNA polymerase that extends nascent RNA-p-DNA strands on the lagging strand template). One difference between *S. cerevisiae* and vertebrates appears to be the role of Mcm10. In *S. cerevisiae*, Mcm10 is essential for the final step of loading Pol-α:primase and Pol-δ, whereas in *Xenopus* egg extracts, Mcm10 is essential for loading Cdc45. In both cases, Mcm10 stabilizes the final replisome structure.

### Preventing DNA re-replication

**Figure 6:**
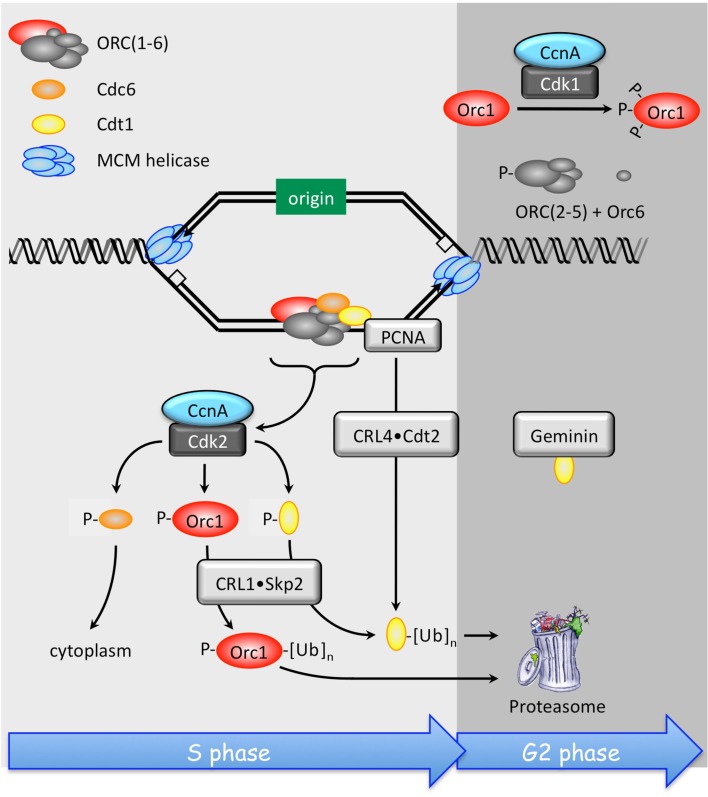
**Multiple concerted pathways prevent DNA re-replication in mammals**. Once mammalian cells have entered S phase, re-replication of DNA that has already been replicated is prevented by inactivation of Orc1, Cdc6, and Cdt1 by direct Cdk2•CcnA dependent phosphorylation. In addition, Cdc6-P is localized to the cytoplasm, and both Orc1-P and Cdt1-P are substrates for the ubiquitin ligase CRL1•Skp2. Cdt1 that is not phosphorylated, but associated with PCNA and DNA, is targeted for degradation by the CRL4•Cdt2 ubiquitin ligase. PCNA is the eukaryotic sliding clamp protein that allows DNA polymerases-δ and -γ to synthesize DNA continuously without falling off the primer–template. Binding of Geminin also inhibits Cdt1, and Orc1 is also inhibited directly by Cdk1•CcnA phosphorylation, events that are required during G2 and early M phase.

Restricting genome duplication to once per cell division requires that cells do not reinitiate nuclear DNA replication within regions that have already been replicated until cell division is completed. Mammalian cells accomplish this through multiple concerted pathways (Figure 6; DePamphilis et al., 2006; Arias and Walter, 2007). As mammalian cells enter S phase, each DNA replication bubble contains ORC, Cdc6, and Cdt1 bound to one copy of the replication origin, whereas the other copy does not. In addition, many preRCs are found at dormant replication origins that are activated only under conditions of replication stress, such as reduction of dNTP pools (Blow et al., 2011). Therefore, to prevent reinitiation of nuclear DNA replication, the cell must inactivate both bound and unbound preRC proteins.

The first line of defense is Cdk2•CcnA. This protein kinase phosphorylates Orc1, Cdc6, and Cdt1, thereby suppressing their activities and preventing their participation in further initiation events. Phosphorylated Cdc6 (Cdc6-P) is localized to the cytoplasm where it cannot participate in origin licensing. The ubiquitin ligase CRL1/SCF•Skp2 targets Orc1-P and Cdt1-P for export to the cytoplasm where they are degraded by the 26S proteasome (Saxena and Dutta, 2005; Saha et al., 2006). Orc1 inactivation is followed by subsequent release of the other ORC subunits (Siddiqui and Stillman, 2007). Only the Orc1, Orc2, and Orc6 subunits contain potential CDK phosphorylation sites, and CDK-dependent phosphorylation of Orc2 destabilizes the remaining ORC subunits (Lee et al., 2012b). This S phase dependent release of ORC(1–6) from chromatin occurs also in flies, frogs, and worms (Rowles et al., 1999; Sun et al., 2002; Remus et al., 2005; Sonneville et al., 2012). In addition, CRL4•Cdt2 inactivates preRCs associated with chromatin by ubiquitinating non-phosphorylated Cdt1 that is bound to both PCNA and DNA (Havens and Walter, 2011). Degradation of Cdt1 by CRL4•Cdt2 occurs in fungi and metazoa, whereas CRL1•Skp2 mediated Cdt1 degradation has been demonstrated only in humans; the pathway is not conserved in yeast, invertebrates, or even among other vertebrates (Kim and Kipreos, 2007).

As cells enter G2 and M phases, preRC proteins are again expressed but in modified forms that both suppress their activity and prevent their destruction. The largest ORC subunit, Orc1, is hyperphosphorylated by Cdk1•CcnA (Li et al., 2004) and transported to the cytoplasm (Saha et al., 2006). Thus, CDK dependent phosphorylation prevents both Orc1 and Cdc6 from initiating preRC assembly until cells exit metaphase. Moreover, phosphorylation protects Cdc6 from being targeted for degradation by the APC (Mailand and Diffley, 2005). The same may be true for other preRC components. Geminin binds to and inactivates Cdt1, thereby preventing it from loading MCM helicases onto ORC•Cdc6•chromatin sites during the G2 to M phase transition (Klotz-Noack et al., 2012). As with phosphorylation of Cdc6, Geminin also protects Cdt1 from ubiquitin-mediated degradation (Ballabeni et al., 2004). As cells exit metaphase, Cdc14 phosphatase dephosphorylates the ORC subunits and Cdc6, making them available for preRC assembly in the absence of CDK activity (Zhai et al., 2010). Geminin is targeted by the APC for ubiquitin-mediated degradation, thereby making Cdt1 immediately available to complete preRC assembly by recruiting the MCM helicase.

In addition to these safeguards, DNA replication stress, such as reduction of dNTP pools, activates the DNA replication checkpoint pathway. The effector kinase of this pathway, Chk1, inhibits activation of preRCs in two ways: Chk1 inhibits Cdc25 phosphatases by phosphorylating them at specific sites, thereby preventing activation of Cdk2 and Cdk1 (Figure 4; reviewed in Chen and Poon, 2008). This prevents activation of preRCs, because Cdk1 is the only CDK that can substitute for Cdk2 in regulating S phase (discussed below). Chk1 also phosphorylates Treslin/Sld3, thereby preventing the interaction between Treslin/Sld3 and TopBP1/Dpb11. This prevents formation of the Cdc45•MCM•GINS complex that is essential for migration of replication forks (Boos et al., 2011).

### Inducing DNA re-replication selectively kills cancer cells

Of the eight pathways that prevent DNA re-replication (Figure 6), six are CcnA dependent, one is Geminin dependent, and one is CRL4 dependent. Suppressing expression of CcnA or CRL4 activity with siRNAs can induce marginal amounts of DNA re-replication in human cells derived from either normal or cancer tissues (Lovejoy et al., 2006; Machida and Dutta, 2007; Zhu and Depamphilis, 2009). However, human cancer cells are far more dependent than normal cells on Geminin to regulate origin licensing (Zhu and Depamphilis, 2009). Suppression of Geminin alone is sufficient to induce extensive DNA re-replication in cells derived from human cancers, but not in cells derived from normal tissues. Inducing extensive DNA re-replication in cells derived from normal tissues requires suppression of both CcnA and Geminin. This distinction might account for the genetic instability commonly seen in cancer cells as a consequence of inadequate regulation of origin licensing, which leads either to under- or over-replication of chromosomal DNA (Blow and Gillespie, 2008). Suppression of Gmnn expression by siRNA in cells derived from a variety of carcinomas, adenocarcinomas, glioblastomas, and osteosarcomas induces DNA re-replication, DNA damage, a DNA damage response, and finally apoptosis (Melixetian et al., 2004; Zhu et al., 2004; Tachibana et al., 2005; Zhu and Dutta, 2006), whereas under the same conditions, suppression of Geminin in cells derived from normal human tissues does not prevent their continued proliferation (Zhu and Depamphilis, 2009). This distinction is not an experimental artifact, because DNA re-replication could be induced in normal cells if both Geminin and CcnA expression are suppressed, either directly with individual siRNAs (Zhu and Depamphilis, 2009), or indirectly by suppressing expression of Emi1 (Di Fiore and Pines, 2007; Machida and Dutta, 2007; Lee et al., 2012a). Emi1 binds to and inhibits the APC during S, G2, and early M phases, thereby preventing premature activation of the APC with concomitant destruction of CcnA, CcnB, and Geminin. Therefore, in many cancer cells, Geminin is essential to prevent DNA re-replication, whereas in normal cells, both Geminin and one or more of the CcnA dependent pathways prevent DNA re-replication (Figure 6). Thus, suppressing Geminin activity in humans could selectively kill a variety of different cancer cells with little or no effect on proliferation of non-cancerous tissues. Candidates for such drugs are currently being identified by high throughput screens of small molecules for compounds that mimic the effects of siRNA against Geminin on human cells (Zhu et al., 2011; Lee et al., 2012a).

## How Protein Kinases Control DNA Replication

The eight protein kinases that directly control DNA replication during cell division can be classified into three groups: Dbf4-dependent proteins kinases, CDKs, and checkpoint kinases. What exactly do these proteins do?

### The role of DDK enzymes

The Dbf4-dependent protein kinases are composed of a catalytic subunit (Cdc7) and a regulatory subunit (Dbf4 or Drf1). DDK is involved in replisome assembly, loading of cohesins onto chromatin, phosphorylation of the checkpoint mediator protein claspin, and the activation of the ATR–Chk1–Cdc25 DNA replication checkpoint (Swords et al., 2010). Not surprisingly, Cdc7 is required for normal mouse development. Studies in yeast and in frog egg extracts suggest that DDK is recruited to replication origins by the MCM helicase where it promotes the stable formation of the Cdc45•MCM helicase complex by phosphorylating each of the subunits in the MCM helicase, except for Mcm5 (Labib, 2010; Sheu and Stillman, 2010; Heller et al., 2011).

### The role of CDK enzymes

As with DDK enzymes, the cyclin-dependent protein kinases consist of a catalytic subunit (Cdk) and a regulatory subunit (cyclin) that is essential for CDK activity and target specificity. In yeast, a single Cdk subunit (termed either Cdk1, Cdc28, or Cdc2) regulates cell division by pairing with five to seven different cyclin subunits. The human genome encodes 13 different Cdk subunits, of which 11 have been characterized, and 25 cyclins, of which 19 have been characterized. However, only Cdk1, Cdk2, Cdk4, and Cdk6 are involved directly in driving the mitotic cell cycle. They are activated by only 10 different cyclins from four gene families: cyclins A, B, D, and E. However, these CDK•cyclin complexes are not active unless the Cdk subunit is phosphorylated at a specific site by Cdk7•CcnH. Remarkably, not all of these proteins are essential for mammalian development, as other members of the Cdk and cyclin families can play some of their roles.

Studies with frog egg extracts and mammalian cells make a compelling case that Cdk2 is required to initiate and regulate S phase in multicellular eukaryotes (see Berthet et al., 2003; Ortega et al., 2003). However, Cdk2 is dispensable for the regulation of mitotic cell cycles in mice as long as both Cdk1 and Cdk4 are available (reviewed in Satyanarayana and Kaldis, 2009). As with yeast, Cdk1 alone can drive the mammalian cell cycle, by replacing Cdk2 in the initiation and regulation of S phase. Other than Cdk1, ablation of individual mouse CDKs has little effect on cell cycle progression and mouse embryonic development (Table 2). Mouse embryos lacking Cdk2, Cdk3, Cdk4, or Cdk6 develop into viable adults with only mild delays in cell cycle progression. Deletion of both Cdk4 and Cdk6 do not significantly affect mouse development or proliferation of mouse embryonic fibroblasts. Even simultaneous deletion of Cdk2, Cdk4, and Cdk6 allows embryos to develop up to midgestation (gestation occurs from 19 to 21 days after fertilization), and embryonic fibroblasts lacking Cdks 2, 4, and 6 still proliferate, although more slowly, and still develop into immortalized cell lines. Thus, Cdk1 alone can substitute for Cdks 2, 4, and 6 through trillions of cell divisions. At that point, a specific requirement of either Cdk4 or Cdk6 for hematopoiesis results in embryonic death due to anemia. Clearly, the mitotic cell cycle is highly conserved throughout the eukarya, and the multitude of CDKs encoded by mammals evolved to carry out specific tasks required in the development of complex multicellular organisms.

**Table 2:** **Effect of CDK deletions on mouse development**.

Deletion	Phenotype	Reference
Cdk1	Embryonic lethality during first cell divisions	Santamaria et al. (2007)
Cdk2	Reduced body size, impaired neural progenitor cell proliferation, infertility in males and females	Berthet et al. (2003), Ortega et al. (2003)
Cdk3	Viable and fertile	Ye et al. (2001)
Cdk4	Reduced body size, insulin deficient diabetes, infertility in males and females	Rane et al. (1999), Tsutsui et al. (1999)
Cdk5	Death at birth, severe neurological defects	Ohshima et al. (1996)
Cdk6	Fertile hypoplasia of thymus and spleen, defects in hematopoiesis	Malumbres et al. (2004)
Cdk4 + Cdk6	Lethality between embryonic day E14.5 and birth. Defective hematopoiesis, MEFs are viable	Malumbres et al. (2004)
Cdk2 + Cdk4	Lethality between E14.5 and E16.5, cardiac and hematopoietic abnormalities	Berthet et al. (2006)
Cdk2 + Cdk6	Infertility in males and females, defective hematopoiesis	Malumbres et al. (2004)
Cdk2 + Cdk4 + Cdk6	Lethality by E13.5 due to hematopoietic defects. MEFs proliferate slowly, but become immortal	Santamaria et al. (2007)

The CDK regulatory subunits also exhibit functional redundancy. As with Cdk4 and Cdk6, ablation of any one of their cyclin partners (the CcnD1, CcnD2, or CcnD3 gene) does not affect mouse development, viability, or fertility (Table 3). Therefore, Cdk4 and Cdk6 can use any one of the three cyclin D proteins. Ablation of all three cyclin D genes also does not affect the ability of mouse embryonic fibroblasts to proliferate, thereby confirming that the functions of Cdk4•CcnD and Cdk6•CcnD are not essential for cell proliferation. However, mouse embryos lacking all three cyclin D genes die at E16.5 due to proliferative defects in hematopoietic cells and cardiac myocytes (Table 3), which reveals that the role of these enzymes is required for specific developmental events. That role may reside in the “G1 restriction point” that prevents preRCs from becoming preICs in the absence of nutrients or mitogens (Figure 2A; DePamphilis and Bell, 2011). The G1 restriction point marks the transition from an inactive form of the transcription factor E2f (the E2f•Rb complex) to an active form of E2f. This transition occurs when Rb1 is phosphorylated by Cdk4,6•CcnD (Figure 3). E2f then activates transcription of genes such as CcnE that are required to initiate and maintain S phase. Both Cdk4,6•CcnD and Cdk2•CcnE inactivate the CDK-specific inhibitor p27 whose role is to prevent premature entrance into S phase (Figure 2A), although by totally different mechanisms.

**Table 3:** **Effect of cyclin deletions on mouse development**.

Deletion	Phenotype	Reference
CcnA1	Viable, infertile males	Liu et al. (1998)
CcnA2	Death by E5.5	Murphy et al. (1997), Kalaszczynska et al. (2009)
CcnB1	Death by E10.5	Brandeis et al. (1998)
CcnB2	Viable and fertile	Brandeis et al. (1998), Draviam et al. (2001)
CcnD1 or D2 or D3	Viable and fertile	Ciemerych et al. (2002)
CcnD1 + D2 + D3	Death by E16.5, proliferative defects in hematopoietic cells and cardiac myocytes, viable MEFs	Kozar et al. (2004)
CcnE1 or E2 CcnE1 + E2	Viable and fertile Late embryonic death, rescued using wild-type placenta, no TG endoreplication, viable MEFs	Geng et al. (2003), Parisi et al. (2003)

The requirement for cyclin E follows a pattern similar to that for cyclin D. Ablation of either CcnE1 or CcnE2 does not interfere with normal embryo development, although ablation of both CcnE1 and CcnE2 results in late embryonic death (Geng et al., 2003; Parisi et al., 2003). Death, however, does not result from a failure in mitotic cycles, but from defects in placentation and hematopoiesis. Mouse embryonic fibroblasts that lack CcnE are viable, but endocycles in trophoblast giant (TG) cells and megakaryocytes are severely impaired in the absence of CcnE.

Mammals have two A-type cyclins and two B-type cyclins, but only CcnA2 and CcnB1 are essential for mouse development (Table 3). Cdk2•CcnA2 prevents DNA re-replication during S phase (Figure 6). Cdk1•CcnA2 not only phosphorylates ORC subunits during the G2 to M phase transition (Li et al., 2004), but phosphorylates histone H3 as cells enter prophase, thereby facilitating chromatin condensation (Gong and Ferrell, 2010). CcnA2 also is required for the activation and nuclear localization of Cdk1•CcnB1. CcnB2 is restricted to reorganizing the Golgi apparatus at mitosis, but Cdk1•CcnB1 is the primary force that drives cells from G2 into mitosis (Brandeis et al., 1998; Draviam et al., 2001). Not surprisingly, Cdk1•CcnB1 activity is carefully regulated. Once cyclin B binds to Cdk1, the complex is inactivated by Wee1, a protein kinase that phosphorylates T14 and Y15 in human Cdk1 (Morgan, 2007). Then, in G2 phase human cells, Cdc25 phosphatase converts Cdk1•CcnB1 from an inactive form to an active form, thereby initiating prophase (Gavet and Pines, 2010). For example, Cdk1•CcnB1 phosphorylates the nuclear lamina, triggering breakdown of the nuclear envelope. Cdk1•CcnB1 levels rise to their maximum extent over the course of approximately 30 min, with different levels of CDK kinase activity triggering different mitotic events by selectively phosphorylating a large number of substrates, including the APC.

Before Cdk1, Cdk2, Cdk4, or Cdk6 can form a stable CDK•cyclin complex, the CDK catalytic subunit must be activated by the “CDK-activating kinase.” In flies and vertebrates, this kinase is a complex of Cdk7, cyclin H (CcnH), and Mat1 (Merrick et al., 2008). Mat1 is required only in activating RNA polymerase (Patel and Simon, 2010). Cdk7•CcnH activates the CDK catalytic subunit by phosphorylating threonine residue 160 in the CDK activation loop. This phosphorylation stabilizes the CDK•cyclin complex. Thus, the ability of these four CDKs to function depends on when during the mitotic cell cycle this activation step occurs. In mammalian cells, Cdk7•CcnH phosphorylates Cdk2 before Cdk2 binds a cyclin. This means that Cdk2 forms an active kinase as soon as a cyclin is available. In contrast, Cdk7•CcnH phosphorylates Cdk1 only after it binds a cyclin. Since Cdk1•cyclin complexes are unstable until after Cdk7•CcnH has phosphorylated them, this means that active Cdk1•cyclin complexes cannot form until the concentration of cyclin is great enough for mass action to produce significant amounts of Cdk1•cyclin complex. The competitive advantage for cyclin partners enjoyed by Cdk2 means that even though Cdk1 is ∼10-fold more abundant than Cdk2 in human cells, the appearance of CcnA would strongly favor formation of active Cdk2•CcnA enzyme over active Cdk1•CcnA enzyme.

### The role of checkpoint kinases

The two remaining kinases directly involved in regulation of nuclear DNA replication in mammals are the two closely related checkpoint kinases: Chk1 and Chk2 (reviewed in Chen and Poon, 2008). They are part of the DNA damage response pathway that prevents cells from entering mitosis until they have completed genome duplication. Chk2 is the effector kinase in the Atm–Chk2–Cdc25 pathway that senses double strand DNA breaks (Reinhardt and Yaffe, 2009). Chk2−/− mice are viable and fertile, although sensitive to ionizing radiation (Takai et al., 2002). Therefore, Chk2 is not an essential gene in mammals, apparently because Chk1 can substitute for Chk2.

Checkpoint kinase-1 is essential for mammalian cell proliferation and embryonic development (Liu et al., 2000; Lam et al., 2004; Niida et al., 2005; Tang et al., 2006). It is the effector kinase in the Atr–Chk1-Cdc25 DNA damage checkpoint pathway that senses RPA bound to single-strand DNA breaks, bulky lesions, and stalled replication forks (Smits et al., 2010). The ataxia telangiectasia and Rad3-related (Atr) protein kinase activates Chk1, which then inhibits origin firing (Smits et al., 2010). At low levels of replication stress, Chk1 suppresses origin initiation predominantly by inhibiting the activation of new replication factories (clusters of replisomes identified as replication foci), thereby reducing the number of active factories (Ge and Blow, 2010). At the same time, inhibition of replication fork progression allows dormant origins to initiate within existing replication factories. The checkpoint mediator protein Claspin facilitates the phosphorylation and activation of Chk1 by ATR and therefore is also required for efficient DNA replication. Claspin and Chk1 regulate fork stability and fork density in unperturbed cells as well as in cells suffering from induced DNA damage. However, whereas Chk1 regulates origin firing predominantly by controlling activation of Cdk2 by Cdc25 phosphatase (Figure 4), Claspin plays a role in regulating replication fork stability that is independent of its function in mediating Chk1 phosphorylation (Scorah and McGowan, 2009). The ability of Chk1 to promote replication fork progression on damaged DNA templates relies on its recently identified proliferating cell nuclear antigen-interacting motif, which is required for its release from chromatin after DNA damage (Speroni et al., 2012). Only then is Chk1 kinase activity and interaction with Claspin required for the DNA damage response.

## Initiating Endoreplication

The paradigm for endoreplication in mammals is the differentiation of trophoblast stem (TS) cells into TG cells, an example of developmentally programmed polyploidy in which the DNA content of TG cells *in vivo* can reach >1000C, although TG cells generated *in vitro* seldom exceed 64C (Hu and Cross, 2010). TS cells are derived from the trophectoderm of blastocysts, the outer layer of epithelial cells that give rise exclusively to the various cells comprising the placenta. When re-established in a blastocyst, TS cells contribute exclusively to the placenta, giving rise to all of the cell lineages derived from the trophectoderm (Oda et al., 2010). When cultured *in vitro*, TS cells proliferate in the presence of fibroblast growth factor-4 (FGF4) and conditioned medium, but in the absence of these mitogens, they differentiate into the TG cells essential for implantation and placentation.

Based on studies of mouse TS and TG cells as well as mouse placentation, a mechanism has emerged that is consistent with studies of endoreplication in flies (Ullah et al., 2009b). Cells exit the mitotic cell cycle during the G2 to M phase transition when one S phase has been completed, but the nuclear membrane remains intact and the chromosomes have not undergone condensation. Exit is achieved by inhibiting Cdk1 activity. In mammals, this is accomplished by expression of Cdkn1c/p57/Kip2 (p57) and p21, two CDK-specific inhibitors that are unique to mammals. In flies the same event is accomplished by down-regulating the mitotic activators stg/cdc25, cyclin A and cyclin B as well as up-regulating Dacapo, their only CDK-specific inhibitor.

The critical event in the transition from mitotic cell cycles to endoreplication in both flies and mammals is the switch from a CDK-dependent form of the “APC” (APC•Cdc20/Fzy) to one that functions only in the absence of CDK activity (APC•Cdh1/Fzr; Ullah et al., 2009b). Cdc20 and Cdh1 are the mammalian homologs of the *Drosophila* Fzy and Fzr proteins, respectively. Both APCs ubiquitinate cyclin A, cyclin B, and Geminin, thereby targeting them for degradation by the 26S proteasome, but neither ubiquitinates cyclin E. Since cyclins A and B promote mitosis, and cyclin A and Geminin prevent preRC assembly, inactivation of these proteins promotes the transition from mitosis to G1 phase. This allows replication licensing to occur again without the cell passing through mitosis, and the subsequent rise in cyclin E to activate preIC assembly and trigger a second S phase.

Multiple S phases increase the probability of stalled replication forks and DNA damage, but in flies cells programmed to endoreplicate their genomes do not undergo apoptosis when DNA re-replication is induced by over-expression of Cdt1, whereas cells undergoing mitotic cell division do (Mehrotra et al., 2008). In mouse TG cells, apoptosis due to endocycle induced DNA replication stress appears to be suppressed by the absence of Chk1 protein and the expression of the CDK-specific inhibitor p21.

### The role of p57

Selective inhibition of Cdk1 induces endoreplication in a variety of cells, including those from yeast, flies, birds, and mammals (Ullah et al., 2009b). RO3306 is a drug that can selectively inhibit Cdk1 activity in mammalian cells; thereby arresting them during the G2 to M phase transition and inducing endoreplication in some, but not all, cell types (Vassilev et al., 2006; Ullah et al., 2008; Ma et al., 2009). These differences likely reflect the fact that selective inhibition of Cdk1 activity reversibly arrests cells in G2 phase without inducing endoreplication, whereas cells arrested during prometaphase endoreplicate, because the loss of Cdk1 activity activates the APC which then targets Gmnn, CcnA, and CcnB for degradation (Hochegger et al., 2007). Selective inhibition of Cdk1 by RO3306 in proliferating TS cells not only initiates up to five endocycles, but it also induces differentiation into TG cells. In contrast, the same treatment induces abortive endoreplication and apoptosis in ES cells. Thus, TG cells are unique in that they differentiate into viable, polyploid, non-proliferating cells under conditions in which cells that are not programmed for terminal differentiation undergo apoptosis.

How is Cdk1 activity in TS cells inhibited *in vivo*? FGF4-deprivation of TS cells results in a rapid decrease in Cdk1 activity that cannot be accounted for by corresponding changes in the protein levels of Cdk1 or cyclins A, B, or E (Ullah et al., 2008). The loss of Cdk1 activity is accompanied by a concomitant increase in the expression of p57 and p21, two CDK-specific inhibitors that target Cdk1 and Cdk2 (Ullah et al., 2008). Both p57 and p21 are unique to mammals, and one or both are expressed in all terminally differentiated cells (Ullah et al., 2009a). Expression levels for the third member of this gene family, p27, remain constant during differentiation, presumably because p27 and its orthologs in other eukarya prevent premature entrance into S phase during both mitotic cell cycles and endocycles (Pagano, 2004). In TS cells, the role of RO3306 is played by p57, which is essential for switching from mitotic cell cycles to endocycles. FGF4-deprivation of TS(p57−/−) cells results in several rounds of cell division followed by formation of multinucleated TG cells. Thus, in the absence of p57, loss of the mitogenic stimulus induces differentiation into TG cells in which mitosis can continue in the absence of cytokinesis. This could account for the observed correlation between reduced p57 expression, placentamegaly, and preeclampsia in both mice and humans (Knox and Baker, 2007; Ullah et al., 2008).

### The role of Chk1

What links FGF4-deprivation to expression of p57 and p21? Recent studies have revealed that the *p21*, *p27*, and *p57* genes are transcribed and translated in proliferating TS cells, but p21 and p57 proteins are phosphorylated, thereby selectively targeting them for degradation by the 26S proteasome (Ullah et al., 2011). Remarkably, Chk1, the same protein kinase that prevents cells from entering mitosis before the completion of S phase, also prevents TS cells from premature differentiation into TG cells. Chk2 cannot substitute for Chk1 in suppressing expression of the *p57* and *p21* genes.

Fibroblast growth factor-4-deprivation of TS cells selectively suppresses expression of Chk1 protein with concomitant induction of both p57 and p21 proteins and differentiation of TS cells into TG cells. These two events are linked directly, because either siRNA suppression of Chk1 protein expression, or chemical inhibition of Chk1 kinase activity in proliferating TS cells induces expression of p57 and p21 proteins (Ullah et al., 2011). Conversely, ectopic expression of Chk1 kinase activity in TG cells suppresses expression of p57 during G phase, thereby restoring mitosis – but not cytokinesis – and the appearance of multinucleated cells. The mechanism by which Chk1 expression is suppressed in response to FGF4-deprivation of TS cells remains to be elucidated, although it likely involves one of the ubiquitin ligases that target this enzyme (Leung-Pineda et al., 2009; Zhang et al., 2009).

**Figure 7:**
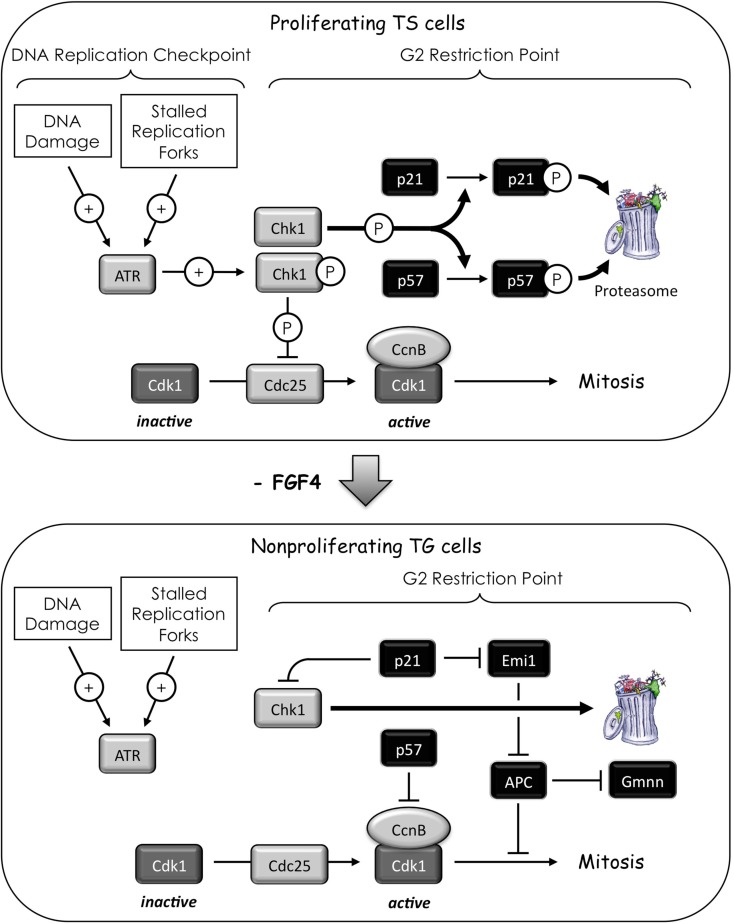
**Activating endocycles in the mouse trophectoderm lineage**. Chk1 is the effector kinase for two checkpoints. The “DNA replication checkpoint” prevents premature mitosis in response to DNA damage and stalled replication forks. These events activate (+) the ATR kinase that activates the Chk1 kinase that inhibits (⦹) the CDC25 phosphatase through site specific phosphorylation. Since CDC25 is required to activate Cdk1, inhibition of CDC25 prevents entrance into mitosis. The “G2-restriction point” prevents expression of p57 and p21 in response to mitogen stimulation. In the case of trophoblast stem (TS) cells, the mitogen is FGF4. In the presence of FGF4, TS cells express the p21 and p57 genes, but Chk1 phosphorylates the p21 and p57 proteins, thereby targeting them for degradation by the 26S proteasome. This prevents TS cells from exiting their mitotic cell cycle. In the absence of FGF4, Chk1 protein is degraded, thereby up-regulating p57 and p21. A feedback loop exists in which p21 could sustain suppression of Chk1 expression in trophoblast giant (TG) cells (Gottifredi et al., 2001). Inhibition of Cdk1 by p57 triggers endoreplication and differentiation into TG cells. This event could be facilitated by p21-dependent down-regulation of Emi1, a specific inhibitor of the APC (Lee et al., 2009). The APC targets mitotic cyclins and Geminin for degradation, thereby promoting origin licensing. The absence of Chk1 in TG cells allows p57 protein levels to oscillate. As CcnE levels increase, Cdk2•CcnE will eventually phosphorylate p57 at a CDK-specific site, thereby targeting it for degradation.

Thus, in the absence of induced DNA damage, Chk1 serves as a mitogen-dependent protein kinase that prevents proliferating TS cells from exiting their mitotic cell cycle and differentiating into TG cells. This means that the same effector kinase that arrests cell proliferation in response to DNA damage and stalled replication forks (“DNA Replication Checkpoint”) prevents the arrest of cell proliferation during the G2 to M phase transition in the absence of induced DNA damage (“G2 Restriction Point,” Figure 7). Small wonder then that the *Chk1* gene is essential for cell proliferation, and that Chk1 deficiency results in peri-implantation embryonic lethality (Liu et al., 2000; Lam et al., 2004; Niida et al., 2005; Tang et al., 2006). Moreover, since the ability of Chk1 to prevent p57 expression is not restricted to TS cells, the G2 Restriction Point presumably exists in all cells that are developmentally programmed for terminal differentiation by up-regulating either p57 or p21. Hence, pluripotent cells in preimplantation embryos can transcribe their *p57* and *p21* genes as soon as the trophectoderm lineage is specified at the eight-cell stage (Yagi et al., 2007), but Chk1 prevents their differentiation into giant cells until the mural trophectoderm is deprived of FGF4 during the peri-implantation period. The G2 restriction point likely operates throughout mammalian development to prevent premature cell cycle exit in progenitor cells destined for terminal differentiation.

In contrast to TS cells, suppressing Chk1 activity in cells that do not express their p57 gene results in apoptosis, a consequence of mitotic catastrophe due to accumulated DNA damage and stalled replication forks. Similarly, ablation of the p57 gene in mice delays differentiation during mouse development and increases the frequency of apoptosis (Yan et al., 1997; Zhang et al., 1997). Cells with DNA damage or incomplete genome duplication cannot proceed into mitosis without inducing apoptosis. The Chk1-dependent phosphorylation of Cdc25 prevents mitotic catastrophe during cell proliferation. However, by preventing mitosis in TG cells with p57 and the eventual loss of Cdk1, suppression of Chk1 disrupts the link between the DNA damage response and apoptosis.

### The role of p21

Late replicating heterochromatic sequences in *Drosophila* are under-represented in polyploid cells, apparently because they lack a cell cycle checkpoint for detecting stalled replication forks and DNA damage (Lilly and Spradling, 1996). Similar mechanisms presumably operate in mammals, as well. Although p21 is not essential for either cell proliferation or viability, mammalian cells lacking p21 fail to arrest in G1 phase in response to induced DNA damage (Brugarolas et al., 1995; Deng et al., 1995). In addition to direct inhibition of Cdk1 activity (Satyanarayana et al., 2008), p21 participates in the maintenance of G2 arrest after DNA damage by down-regulating Emi1 in cells that have initiated transient G2 arrest through the ATM/ATR pathway (Lee et al., 2009; Figure 7). Therefore, low levels of p21 may be sufficient to facilitate mitotic cell cycles, whereas high levels of p21 may facilitate terminal cell differentiation by suppressing apoptosis (Ullah et al., 2008). The p21 protein also may act as a feedback loop in the G2 restriction point by suppressing expression of Chk1 (Figure 7). As the level of Chk1 protein diminishes during FGF4-deprivation, the level of p21 increases, and this increase in p21 can further reduce Chk1 mRNA levels (Gottifredi et al., 2001).

## Sustaining Endoreplication (Endocycles)

Once cells exit the mitotic cell cycle and initiate either endoreplication or endomitosis, they are capable of undergoing several additional S phases in the absence of an intervening mitosis, cytokinesis, or apoptosis. How are these multiple S phases in the absence of cell division (termed “endocycles”) sustained?

### The requirement for Cdk2 and cyclin E

In vertebrates, either Cdk2 or Cdk1 is essential to initiate S phase during mitotic cell cycles, but Cdk2 is essential for endoreplication in TG cells, because Cdk1 activity is selectively suppressed (Ullah et al., 2008). Chemical inhibition of both Cdk2 and Cdk1 prevents both TS cell proliferation and differentiation, whereas selective inhibition of Cdk1 prevents proliferation and triggers differentiation. However, *Cdk2*−/− TS cells fail to endoreduplicate when Cdk1 is selectively inhibited by RO3306, whereas they endoreduplicate and differentiate into TG cells when deprived of FGF4. Therefore, p57 levels in TG cells must oscillate in order to allow preRC assembly during G phase followed by CDK-dependent initiation of S phase. In fact, p57 is expressed in TG cells during G phase but not during S phase (Hattori et al., 2000; Ullah et al., 2008).

**Figure 8:**
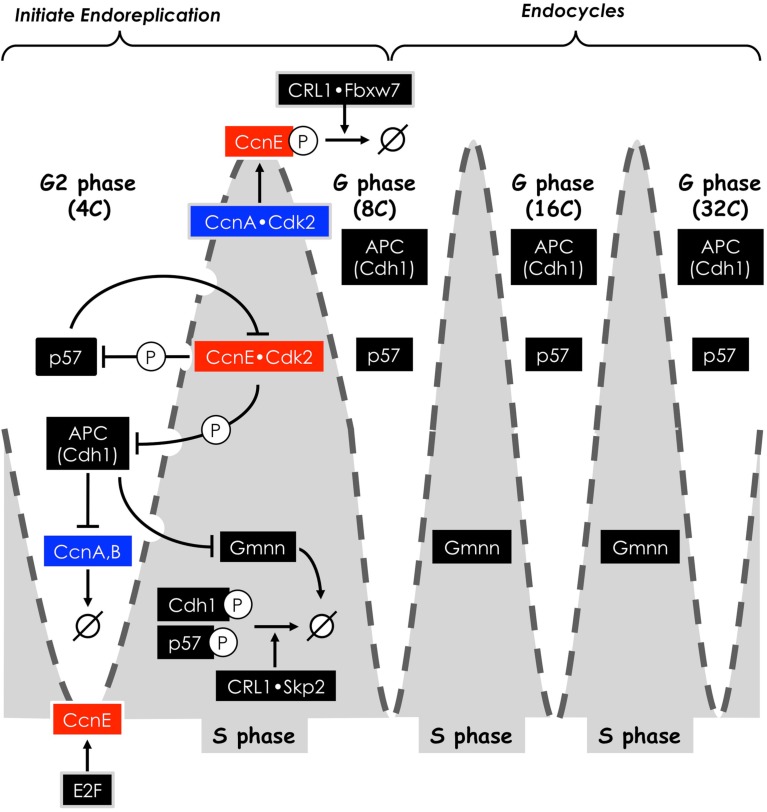
**Sustaining endocycles in the mouse trophectoderm lineage**. Oscillation of APC activity and the levels of CDK-specific inhibitors and Geminin (Gmnn) are inversely related to oscillation of cyclin E (broken gray line). APC activity and CDK inhibitor levels are high in G phases but low in S phases, whereas cyclin E is low in G phases but high in S phases. Cdk2•CcnE activity is required to begin S phase. Endocycles result from a sequence of feedback loops, resulting from phosphorylation events by Cdk2•CcnE, and ubiquitination events by CRL1 and the APC. Ubiquitination by CRL1 requires prior CDK-dependent phosphorylation of its substrate. These events inhibit the activity of their protein targets and cause them to be degraded by the 26S proteasome (→ø). Protein names are those for mammals.

Up-regulation of cyclin E in late G1 phase activates Cdk2, which phosphorylates RecQ4 and Treslin (Table 1; Figure 5), thereby driving the transition from G1 to S phase through assembly of preICs. Thus, premature expression of APC•Cdh1 activity triggers endoreplication by simultaneously preventing mitosis under conditions that allow preRC assembly. Once this occurs, the molecular events that initiate and maintained endocycles in G phase arrested cells (Figure 8) appear to be the same as those that initiate and maintain S phase in mitotic cell cycles (Figures 3 and 4). They are both driven by a series of feedback loops in which phosphorylation by CDK activities, ubiquitination by E3 ubiquitin ligases, and inhibition of CDK activities by specific inhibitors arrest activities in one phase in order to allow new activities to drive the cell into the next phase. Opposing oscillations in cyclin E levels and APC activity allow assembly of preRCs followed by a new round of genome duplication.

### Periodic oscillations in S phase protein kinases

As with mitotic cell cycles, periodic oscillations in protein kinase activities involve the APC (Figure 3). However, genetic and biochemical data from studies on *Drosophila* demonstrate that only APC•Fzr (Fzr is the fly homolog of Cdh1) is essential for endocycles (Sigrist and Lehner, 1997; Schaeffer et al., 2004; Garci-Higuera et al., 2008). The activities of both APC•Fzr and Cdk2•Cyclin E oscillate during endocycles in flies, and these oscillations appear to be driven by changes in Cyclin E levels. Of critical importance is the fact that Cdk2•Cyclin E inactivates Fzr (Sigrist and Lehner, 1997), suggesting that APC•Fzr oscillations are driven by periodic inhibition of Fzr by Cdk2•Cyclin E (Narbonne-Reveau et al., 2008; Zielke et al., 2008). Low Cdk2•Cyclin E activity allows high APC•Fzr activity, which in turn, degrades mitotic cyclins and Geminin, thereby allowing assembly of preRCs. High Cdk2•Cyclin E activity inactivates APC•Fzr, thereby allowing the onset of S phase in the presence of Geminin (Zielke et al., 2008). In addition, the CDK-specific inhibitor Dacapo (the fly homolog of p27) promotes replication licensing during *Drosophila* endocycles by reinforcing low CDK activity during G phase, a role that it appears to play during mitotic cell cycles as well (Hong et al., 2007). The reiteration of these events, driven by the oscillation of Cdk2•Cyclin E, likely drives the progression of endocycles (Narbonne-Reveau et al., 2008; Zielke et al., 2008). Based on mammalian mitotic cell cycles, changes in Cyclin E levels are likely driven by changes in Cdk2•Cyclin A activity, although this remains to be shown.

Mammals appear to sustain endocycles in the same manner as flies, but with some additional detail (Ullah et al., 2009b). In *Drosophila*, Cyclin E is required to drive S phase in both mitotic cell cycles and endocycles, whereas in mammals CcnE is required only for endocycles (Geng et al., 2003; Parisi et al., 2003). In *Drosophila*, endocycles occur under conditions where mitotic cyclins A and B are suppressed, whereas the level of CcnB1 in TG cells remains unchanged from that in TS cells, and the levels of CcnA2 and CcnE1 decrease markedly after the first endocycle (Ullah et al., 2008). Of the nine human CDKs, only Cdk1 can substitute for Cdk2 in driving endocycle S phases (Ullah et al., 2008). Whether or not the same is true in flies remains to be determined. As with flies, oscillation of APC•Cdh1 activity is essential for endocycles in mice (Garci-Higuera et al., 2008; Ma et al., 2009). Thus, during G phase, APC•Cdh1 and p57 (and presumably p27, the mammalian ortholog of Dacapo) suppress CDK activity to allow preRC assembly. Cdk2•CcnE also phosphorylates Cdc6, thereby protecting Cdc6 from APC-dependent proteolysis until it is dephosphorylated and assembled into preRCs (Mailand and Diffley, 2005). The appearance of Cdk2•CcnE activity triggers S phase with concomitant synthesis of Geminin. Geminin and Cdk2•CcnA activity are both required to suppress DNA re-replication in mitotic cell cycles (Zhu and Depamphilis, 2009) and presumably in endocycles as well. Moreover, Cdk2•CcnA phosphorylates CcnE, thereby enabling CRL1•Skp2 to target CcnE for ubiquitin-dependent degradation. In the absence of CcnE, APC•Cdh1 reappears and mediates ubiquitin-dependent degradation of Geminin. In the absence of Cdk1 activity, oscillation of APC activity also requires its phosphorylation by Cdk2•CcnA (Ma et al., 2009).

### The role of p57

Endocycles in TG cells require that p57 levels oscillate to allow preRC assembly in the absence of CDK activity (G phase) followed by their conversion into preICs in the presence of CDK activity (S phase). Thus, suppression of Chk1 activity in TG cells allows expression of p57 during G phase (Ullah et al., 2011), whereas the presence of Cdk2•CcnE activity in TG cells allows p57 to be targeted for degradation during S phase (Hattori et al., 2000; Kamura et al., 2003). Selective degradation of CcnE allows the level of p57 to again rise due to the absence of Chk1 and the loss of Cdk2•CcnE activity, and the cell returns to G phase (Figure 8).

### The role of geminin

Genetic ablation of Geminin in mouse embryos causes excess DNA replication in eight-cell embryos and expression of trophoblast specific genes, suggesting that suppression of Geminin triggers endoreplication and differentiation of pluripotent blastomeres into trophectoderm (Gonzalez et al., 2006). Moreover, silencing Geminin in mouse embryonal carcinoma and ES cells can result in loss of stem cell identity and differentiation into trophoblast cells (Yang et al., 2011). However, others have reported that genetic ablation of Geminin induces DNA re-replication and apoptosis in eight-cell embryos (Hara et al., 2006), and that suppression of Geminin expression does not affect the ability of ES cells to maintain or exit pluripotency (Yellajoshyula et al., 2011). Moreover, expression of Geminin is not suppressed during differentiation of TS cells into TG cells (Ullah et al., 2008), and Geminin levels in *Drosophila* continue to oscillate during endocycles as they do during mitotic cell cycles (Zielke et al., 2008). The simplest explanation is that Geminin prevents DNA re-replication during both mitotic cell cycles and endocycles. Changes in gene expression that occur during Geminin depletion appear to be an indirect consequence of the loss of DNA replication control (Kerns et al., 2012).

## Summary

Errors in genome duplication result in the accumulation of mutations that eventually lead to cancer, a disease in which cells continue to proliferate under conditions where normal cells are either quiescent or terminally differentiated (DePamphilis and Bell, 2011). Therefore, the nuclear DNA component is duplicated once but only once each time a cell divides. To this end, genome duplication is tightly regulated by multiple convergent pathways and inextricably linked to mitosis; DNA re-replication results in apoptosis. Remarkably, only eight (Cdk1, Cdk2, Cdk4, Cdk6, Cdk7, Cdc7, Chk1, Chk2) protein kinases orchestrate these events directly, and only four of them (Cdk1, Cdk7, Cdc7, Chk1) are essential for mammalian development. This paucity in control of such critical events in our biology occurs through replaceable parts and functional redundancy. The extreme example is Cdk4 and Cdk6; they are functionally redundant in the G0 to G1 transition regardless of which of three cyclin D proteins binds to them. In contrast, the Cdk1 and Cdk2 play different roles in the mitotic cell cycle depending on whether they associate with cyclin A, B, or E. Cdk1 can replace Cdk2 in S phase, but Cdk2 cannot replace Cdk1 in mitosis. Similarly, Chk1 can replace Chk2, but Chk2 cannot replace Chk1 in either its DNA damage checkpoint role or its role in TS cell differentiation. Given the importance of preventing excess DNA replication during cell division, the fact that some mammalian cells are developmentally programmed to exit their mitotic cell cycle and differentiate into viable polyploid cells is remarkable. Among the eukarya, the most commonly employed mechanism for this event is endoreplication, whereby selective inhibition of Cdk1 activity occurs under conditions that permit re-licensing of replication origins without passing through mitosis or cytokinesis and without undergoing apoptosis. Chk1 activity is also suppressed, at least in the example of TG cells, apparently as part of the anti-apoptosis mechanism. The remaining members of the protein kinase “octet” remain in their normal mitotic cell cycle roles. We suggest that focusing on the proteins that interact with “the octet” will provide critical insight to the regulation of cell division, its relationship with human disease, and novel directions in cancer therapy.

## Conflict of Interest Statement

The authors declare that the research was conducted in the absence of any commercial or financial relationships that could be construed as a potential conflict of interest.

## Research Topic:

From structural to molecular systems biology: experimental and computational approaches to unravel mechanisms of kinase activity regulation in cancer and neurodegeneration

## Topic Editor:

Matteo Barberis, Humboldt University Berlin, Germany; Max Planck Institute for Molecular Genetics, Berlin, Germany
